# The Impact of the Histone Deacetylase Inhibitor—Sodium Butyrate on Complement-Mediated Synapse Loss in a Rat Model of Neonatal Hypoxia–Ischemia

**DOI:** 10.1007/s12035-024-04591-w

**Published:** 2024-11-12

**Authors:** Karolina Ziabska, Magdalena Gewartowska, Malgorzata Frontczak-Baniewicz, Joanna Sypecka, Malgorzata Ziemka-Nalecz

**Affiliations:** 1 NeuroRepair Department, Mossakowski Medical Research Institute, Polish Academy of Sciences, 5 A. Pawinskiego Street, 02-106 Warsaw, Poland; 2 Electron Microscopy Research Unit, Mossakowski Medical Research Institute, Polish Academy of Sciences, 5 A. Pawinskiego Street, 02-106 Warsaw, Poland; 3 Higher School of Engineering and Health in Warsaw, 18 Bitwy Warszawskiej 1920r. Street, 02-366 Warsaw, Poland

**Keywords:** Neonatal Hypoxia–Ischemia, Histone Deacetylase Inhibitors, Sodium Butyrate, Complement System, Synaptic Proteins

## Abstract

Perinatal asphyxia is one of the most important causes of morbidity and mortality in newborns. One of the key pathogenic factors in hypoxic-ischemic (HI) brain injury is the inflammatory reaction including complement system activation. Over-activated complement stimulates cells to release inflammatory molecules and is involved in the post-ischemic degradation of synaptic connections. On the other hand, complement is also involved in regenerative processes. The histone deacetylase inhibitor (HDACi)—sodium butyrate (SB)—provides reduction of inflammation by decreasing the expression of the proinflammatory factors. The main purpose of this study was to examine the effect of SB treatment on complement activation and synapse elimination after HI. Neonatal HI was induced in Wistar rats pups by unilateral ligation of the common carotid artery followed by 60-min hypoxia (7.6% O2). SB (300 mg/kg) was administered on a 5-day regimen. Our study has shown decreased levels of synapsin I, synaptophysin, and PSD-95 in the hypoxic-ischemic hemisphere, indicating synaptic loss after neonatal HI. Transmission electron microscopy revealed injury of the synaptic structures in the brain after HI. SB treatment increased the level of the synaptic proteins, improved tissue ultrastructure, and reduced degradation of the synapses. Neonatal HI induced mRNA expression of the complement C1q, C3, C5, and C9, and their receptors C3aR and C5aR. The effect of SB was different depending on the time after induction of hypoxic-ischemic damage. Our study demonstrated that neuroprotective effect of SB may be related to the modulation of complement activity after HI brain injury.

## Introduction

Hypoxic-ischemic encephalopathy (HIE) in neonates remains an essential reason for central nervous system (CNS) damage. In developed countries, perinatal asphyxia affects 1.5 per 1000 newborns born at term and up to 26 per 1000 live births in low-resource countries [1]. In the most severe cases, HIE causes the death of the newborn. In neonates who remain alive, perinatal hypoxia–ischemia can lead to the development of transient or long-lasting neurological disorders, such as cerebral palsy, epilepsy, spastic paresis, or inhibition of intellectual development. The injury of CNS after HIE is a complex process with a contribution of multifold mechanisms and pathways resulting in both early and delayed brain damage. One of the key pathogenic factors of perinatal brain injury is the inflammatory reaction, induced primarily by rapidly activated microglia and recruitment of peripheral leukocytes, including monocytes/macrophages. However, a growing body of studies indicates that the complement system is also an important component of the inflammatory response after hypoxia–ischemia. The complement system is composed of more than 50 different plasma and membrane-associated proteins. Activation of the complement involves a cascade of enzymatic and non-enzymatic reactions leading to the formation of two crucial enzymes, convertase 3 (C3) and convertase 5 (C5), and in the final step to the creation of the membrane attacking complex (MAC). The primary function of the complement system is opsonization—the “tagging” of pathogens and damaged cells by complement proteins, leading to their elimination by macrophages. Nevertheless, MAC can also stimulate cells to release inflammatory molecules. Moreover, C3 and C5 can recruit leucocytes to the sites of injury. After ischemia, complement proteins pass through the damaged blood–brain barrier to the brain, although they can also be locally produced in the CNS, mainly by glial cells. In particular, microglia express high levels of C1q and C3 receptors, which serve a pivotal role in phagocytosis [2–4]. Astrocytes are a major source of the complement C3 [5] and additionally express C4 and C9 proteins [6]. Furthermore, neuronal complement expression of C1q, C2, C3, C4, C5, C6, C7, C8, and C9 proteins as well as C5a and C3a receptors has been reported both in vivo and in vitro [7–9]. The role of the complement system in neonatal brain injury following hypoxia–ischemia is not completely understood. Complement activation and upregulation of complement proteins in the CNS have been shown in rodent models HIE [10]. Furthermore, few available clinical studies conducted on neonates affected by HIE indicated activation of the complement system in their brains and/or cerebral spinal fluid (CSF) [11, 12]. Recent studies indicate that inhibition of the inflammatory reaction, including complement cascade caused by hypoxia–ischemia may be neuroprotective. However, it should be emphasized that depletion of the complement system activity in the immature brain can be a double-edged sword, because complement is also involved in various developmental and regenerative processes such as synaptic pruning and synaptic plasticity [13]. Weak or inactive synapses targeted for elimination are tagged by complement proteins and removed by microglia. Therefore, there is a need to develop a therapeutic strategy that can modulate complement activity after HI without completely blocking its beneficial functions in the developing brain.

There is growing evidence that epigenetic modifications have been associated with the pathogenesis of several diseases including brain ischemia. As epigenetic changes are reversible, they could assist in the development of new therapeutic approaches for the treatment of post-ischemic injury. Acetylation/deacetylation of lysine residues is a key post-translational modification of the proteins responsible for the regulation of critical intracellular pathways. Two classes of nuclear enzymes, histone acetylases (HATs) and histone deacetylases (HDACs), control chromatin remodeling via histone acetylation/deacetylation. HATs catalyze the acetylation of histone lysine residues which provides a more open chromatin structure and enhances gene transcriptional activity. In contrast, deacetylation of lysine residues by HDACs may cause compression of the DNA/histone complex and suppress gene expression. Moreover, HDACs/HATs modify a large number of non-histone proteins which can alter various intracellular processes. In relation to inflammation, acetylation modulates the activity or function of cytokine receptors, nuclear hormone receptors, transcription factors, and intracellular signaling molecules [14]. Several data published in the last decade provided significant evidence that histone deacetylase inhibitors (HDACis) are the promising group of agents that demonstrate neuroprotective and anti-inflammatory action in animal models of brain ischemia. Administration of these compounds after the onset of stroke in adult animals resulted in a significant reduction in the number of microglial cells, suppression of their activation, and inhibition of other inflammatory markers, which in turn led to improved neuropathological outcomes [15, 16]. Our previous study demonstrated that one HDAC inhibitor—sodium butyrate—has a neuroprotective effect in an animal model of neonatal hypoxia–ischemia. This neuroprotective action of SB was at least in part due to a reduction in inflammation induced by hypoxia–ischemia. We observed that administration of SB decreased the number of activated microglial cells in the hypoxic/ischemic hemisphere and promoted the conversion of microglia phenotype from inflammatory M1 to anti-inflammatory M2. Additionally, SB treatment suppressed the production of inflammatory markers IL-1β and chemokine CXCL10, and blocked ischemia-elicited upregulation of COX-2 [17, 18]. Therefore, in the present study, we aimed to examine the effect of SB treatment on the complement system, another important component of inflammatory response after neonatal hypoxia–ischemia. According to our knowledge, the impact of SB on the complement after neonatal HI has not yet been determined to date. We studied the expression of main complement proteins as well as their receptors in the immature brain after HI. Furthermore, as complement over-activation contributes to the post-ischemic loss of synaptic networks, we have also investigated whether SB can prevent synapse degradation in a model of neonatal hypoxia–ischemia. Understanding the effect of SB action on immune pathways may be helpful in developing further neuroprotective strategies to improve outcomes after neonatal hypoxia–ischemia.

## Material and Methods

### Experimental Model of Neonatal Hypoxic-ischemic Brain Injury

All experiments were performed with a protocol authorized by the 2nd Local Ethical Committee for Animal Experiments in Warsaw (authorization no. WAW2/081/2018) in accordance with the EU Directive 2010/63/EU. All experiments and methods were carried out according to relevant regulations and ARRIVE guidelines.

**Fig. 1:**
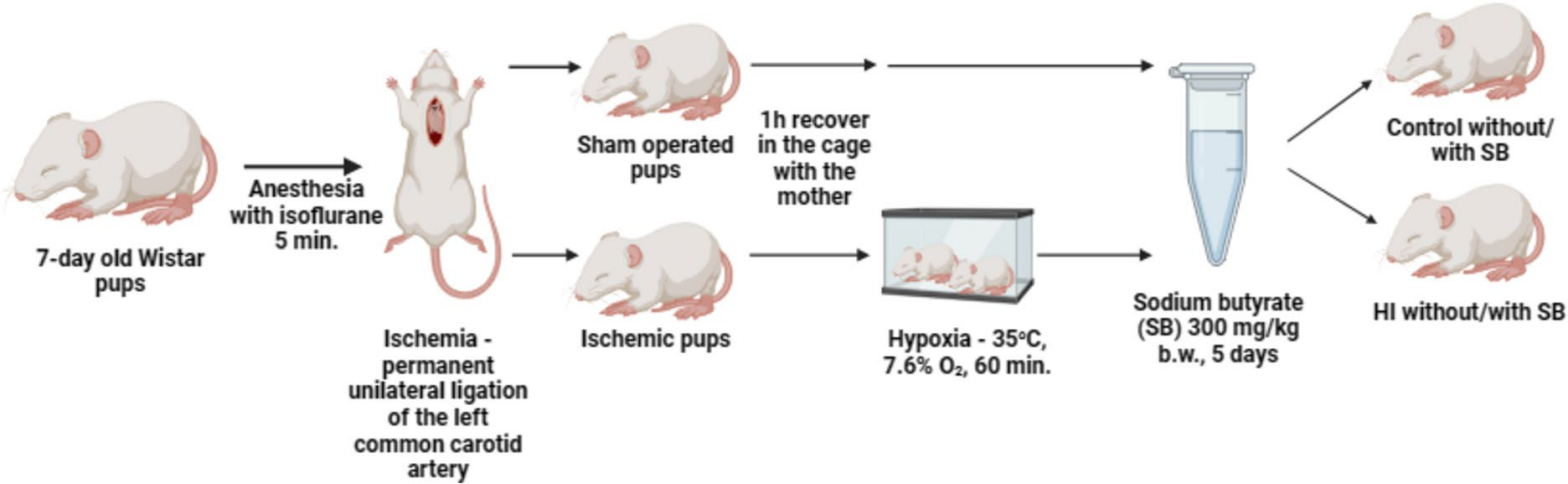
Diagram of the in vivo model. Created with BioRender.com

Neonatal hypoxia–ischemia was induced in Wistar rats of both sexes on postnatal day 7 (P7) by permanent unilateral ligation of the common carotid artery, followed by systemic hypoxia according to the method proposed by Rice et al. [19]. In our previous studies, it was explored if there is a sexually dimorphic response to HI treatment [20]. As we did not notice any significant differences between male and female rats in the previous experiments, pups of either sex were used. Rats from each litter size 10–12 were randomly selected into groups (control or HI). Then, the pups were anaesthetized with isoflurane (4% induction, 2.0% maintenance), and after that, the left common carotid artery was exposed and cut between double ligatures of silk sutures. The incision was sutured and treated with lignocaine. Sham-operated (control) animals will undergo the same surgical procedure without the ligation of the carotid artery. The anesthesia time was no longer than 5 min, to avoid the neuroprotective influence of isoflurane. Then, the rat pups were returned to the mother in the home cage nest for 1 h. After that hypoxia was induced by placing the animals in a chamber at a controlled temperature (35 °C) and subjecting them to a mixture of 7.5% oxygen in nitrogen for 1 h. When the entire procedure was ended, the rats were returned to their home cage housed at 22 ± 2 °C and 55 ± 5% humidity following a 12/12-h light/dark cycle with constant access to food. In this model, hypoxic-ischemic occlusion was observed in the cerebral hemisphere on the side of the ligated artery (ipsilateral), and only these hemispheres were taken to the next experiments. The undamaged hypoxic hemisphere, as well as sham-operated animals, served as controls. The animals were randomly divided into 4 experimental groups according to the following pattern (3–5 rats per group and time point): (1) control animal (non-hypoxic, non-ischemic); (2) control animals treated with sodium butyrate, to assess the incidence of unintended deleterious effects of SB administration in the control groups (Ctr + SB); (3) post-HI animals (HI); (4) post-HI animals treated with sodium butyrate (HI + SB) (Fig. 1). During the experiment, animal mortality did not occur outside of planned endpoint. The brains for further procedures were isolated at 1, 3, 5, 7, and 14 days after HI. For qPCR and Western blot analysis, 64 rats were used (4 groups and 4 time points 1, 3, 5 and 14 days after surgery); for TEM analysis 20 rats were used (4 groups and 1 time point 7 days after surgery): for the immunohistochemical studies 20 rats were used (4 groups and 1 time point 14 days after surgery).

### Pharmacological Stimulation

Sodium butyrate (Sigma-Aldrich) was administered subcutaneously at a dose of 300 mg/kg/day for 5 consecutive days (starting immediately after HI induction) to both control animals (Ctr + SB) and post-HI animals (HI + SB). The optimal doses and timing of sodium butyrate administration were determined in our previous experiments [20].

### Tissue Preparation

At selected time points after HI or controls in match age (7 and 14 days), rats were deeply anaesthetized with intraperitoneal injections of ketamine (100 mg/kg b.w.) combined with xylazine (10 mg/kg b.w.).

For immunohistochemical studies, rats were perfused through the heart with PBS buffer (pH 7.4) followed by a fixative solution of 4% paraformaldehyde in PBS. Then, brains were isolated, postfixed for 3 h at 4 °C in the same solution, cryoprotected in 30% solution of sucrose overnight, and afterward, frozen on dry ice. The hemispheres were then cut (from Bregma − 2.80 mm Lambda 4.80 mm to Bregma 3.40 mm Lambda 4.20 mm) in regions of the cortex and hippocampus into cryostatic slices 30 mm thick, to create 10 serial sections. The brain sections were placed in a 24-well plate with a non-freezing solution (Sigma-Aldrich) and stored at − 20 °C until use.

Molecular (qPCR) and biochemical (Western blot) analyses were performed on non-perfused tissue 1, 3, 5, and 14 days after HI induction or controls in matched age. The animals were anesthetized as described above and decapitated. The hemispheres were dissected, immediately frozen on dry ice, and stored at – 80 °C until use. Only the ipsilateral (hypoxic-ischemic) hemispheres (from HI animals) and the left hemispheres (from control animals) were analyzed.

### Transmission Electron Microscopy

For transmission electron microscopy, the animals were perfused through the heart with PBS buffer (pH 7.4) followed by a mixture of 2.5% glutaraldehyde (GA), 2% PFA, and 0.1 M cacodylate buffer, and then, the brains were isolated and incubated at 4 °C for 24 h in the same solution. After a 24-h incubation, the frontal cerebral cortex (the brain region most affected by HI) was isolated from the brains and cut into small fragments. The tissue then was washed three times in 0.1 M cacodylate buffer and post-fixed in 1% osmium tetroxide (OsO_4_) and 0.8% potassium ferricyanide (K_4_(FeCN)_6_) for 2 h. The tissue was dehydrated in a series of alcohols of increasing concentration (30–99.8%) and in propylene oxide. The samples were then embedded in resin blocks and polymerized at 60 °C for 24 h. The polymerized material was cut into ultra-thin Sects. (40–60 nm) on an MTXL ultramicrotome, using a diamond knife, placed on copper grids (300 mesh) and post-stained using the double contrast method with uranyl acetate (30 min) and lead citrate (15 min). Analysis was performed using a JEM-1011 transmission electron microscope (JEOL) operating at an accelerating voltage of 80 kV. The level of damage to the cytoarchitecture of the cerebral cortex was evaluated, with particular attention to synaptic connections. Transmission electron microscopy studies were performed in cooperation with the Electron Microscopy Research Unit, MMRI PAS.

### Western Blot Analysis

Expression of complement and pre- and postsynaptic proteins were determined by Western blot analysis in brain hemispheres after HI and sodium butyrate treatment. For Western blot analysis, the brain hemispheres were homogenized in a RIPA lysis buffer (10 mM Tris–HCl pH 7.5 containing 150 mM NaCl, 1% Nonidet P40, 0.1% SDS, 1% Triton X-100, PMSF 0.1 mg/mL) supplemented with proteinase and a phosphatase inhibitor cocktail (1:100, Sigma Aldrich). The lysates were centrifuged at 13,000 × *g* for 10 min at 4 °C and the supernatants were collected. Total protein concentrations were assessed using a Bio-Rad DCTM protein assay kit (Bio-Rad). Samples containing 50 μg of protein were separated by SDS–PAGE and then transferred onto nitrocellulose (Amersham™ Protran™ Supported 0.45 μm NC). Each sample was tested at minimum in duplicate and each variant in three biological replicates. After blocking in 5% non-fat milk, the membranes were incubated at 4 °C overnight with primary antibodies. The primary antibodies used for the Western blot analysis: mouse monoclonal anti-PSD-95 (ThermoFisher, 1:2000), mouse monoclonal anti-synaptophysin (Sigma, 1:1000), and rabbit polyclonal anti-synapsin I (ThermoFisher, 1:1000). In the next step of Western blot analysis, the membranes were rinsed 3 times in TBST buffer (1 × Tris-Buffered Saline, 0.1% Tween® 20 Detergent) and then incubated for 1 h at room temperature with an anti-rabbit or anti-mouse horseradish peroxidase-conjugated secondary antibody (Sigma-Aldrich). To verify an equal protein loading per line, a mouse monoclonal anti-β-actin (UniProtKB, 1:1000) was used as an internal control for each Western blot analysis. Immunoblot signals were visualized using an ECL chemiluminescence kit (GE Healthcare Life Sciences) by exposure of the membrane to an X-ray HyperfilmTM ECL film (GE Healthcare Life Sciences). A semiquantitative estimation of protein levels detected by immunoblotting was performed utilizing LKB Utrascan XL Program GelScan software. The densitometry values were averaged in all groups and then the densitometry values in the control groups were taken as 100%. The data from the respective experimental groups are presented as percentages of the control value.

### Immunohistochemical Evaluation

In order to determine the effect of sodium butyrate on the modification of synaptic networks and expression/localization of selected complement proteins in brains after HI injury, double immunohistochemical staining was performed using antibodies labeling selected complement proteins C3 (ThermoFisher/Invitrogen) and C5 (ThermoFisher/Invitrogen) as well as proteins localized in postsynaptic membrane PSD-95 (ThermoFisher/Invitrogen).

The brain sections were placed in a new 24-well plate and washed with PBS. Subsequently, the slices were incubated in a blocking medium (10% normal goat serum in PBS containing 0.1% Triton X-100) for 1 h at room temperature. The sections were incubated overnight at 4 °C with a primary antibody specific for complement proteins (goat polyclonal anti-C3 (1:500) or rabbit polyclonal anti-C5 (1:100)). Subsequently, the slices were rinsed in PBS, exposed to the appropriate Cy3-conjugated secondary antibodies (AlexaFluor 546, 1:500) for 1 h at room temperature in the dark. In the next step of double immunofluorescent staining, sections were washed 3 × 5 min in PBS and incubated with primary antibody for synaptic proteins (mouse monoclonal anti-PSD-95 (1:500)) overnight at 4 °C. Then, after rinsing in PBS, the primary antibodies were revealed by applying the appropriate secondary FITC-conjugated antibodies (AlexaFluor 488, 1:500) for 1 h at room temperature. After washing the slices in PBS (3 × 5 min.), the nuclei were labeled with the fluorescent dye Hoechst 33258 (2 μg/ml PBS; Sigma–Aldrich) for 20 min and washed in PBS. Finally, sections were removed from the wells, applied to microscope slides, coated with Fluorescent Mounting Medium (Dako), and microscope cover glasses, and then dried. Labeling was verified using a confocal laser scanning microscope (LSM 780, Carl Zeiss, Germany) with ZEN software. A helium–neon laser (543 nm) was utilized in the excitation of Alexa Fluor 546, while an argon laser (488 nm) was applied in the excitation of FITC. Images were collected from three different fields of view.

### Reverse Transcription and Quantitative PCR Analysis

The effect of sodium butyrate on gene expression of selected complement proteins (C1q, C3, C5, C9) and their receptors (C3aR, C5aR) was determined in brains of HI-treated and control animals in different experimental time points (24 h, 72 h, and 5 days after surgery procedure).

Total RNA was isolated using the Total RNA Mini Kit (A&A Biotechnology), according to the manufacturer’s instructions. The quality and concentration of RNA were verified by spectrophotometry with the Nanodrop™ apparatus. The samples containing 1 μg of total RNA were reverse transcripted using high-capacity RNA-to-cDNA Kit (Applied Biosystems) according to the manufacturer’s recommendation. The analysis of changes in the mRNA level of genes was carried out using FAST SYBR Green Master Mix Reagent (Applied Biosystems), 2 μl of cDNA samples, and specifically designed primers (Table 1). The quantitative PCR reaction was performed in the 7500 Fast real-time PCR System (Applied Biosystems). The reaction steps were as follows: (1) holding stage, 10 s at 95 °C; (2) cycling stage (40 ×), 3 s at 95 °C, 30 s at 60 °C, and 45 s at 72 °C; and (3) melt curve stage, 15 s at 95 °C, 1 min at 60 °C, repeated in two cycles. Each sample was tested in triplicate, and each variant was in three biological replicates. The dissociation curve will be plotted to determine the specificity of the amplification. The fluorescence signals of a specific transcript were normalized against those of the reference gene (β-actin), and the threshold cycle values (ΔCt) were quantified as fold changes using the 2^−ΔΔCT^ method.

**Table 1:** List of designed primers used in quantitative real-time PCR analysis

Gene *starter*	Sequence (5’-3’)	Melting temperature (T_M_) [°C]
C1qa *forward*	GCTAACACCTGAAAGAGCCC	53,8
C1qa *reverse*	GTGATCTCCAAGTGGGACCT	53,8
C3 *forward*	CGCACTGTTTCTGGTTCTTCTG	54.8
C3 *reverse*	AGGTCAGGAACGAAGGTTCATC	54.8
C5 *forward*	CAGCATAATTCAGGGTGAACG	52.4
C5 *reverse*	CAGCTTGTCATTTGAGCCAC	51.8
C9 *forward*	TTTATTAACGAGAGATTGGCATTG	50.6
C9 *reverse*	TGATGTCGTCTCTGTTTCAGGTC	55.3
C3aR f*orward*	GACCTACACTCAGGGC	48.5
C3aR *reverse*	ATGACGGACGGGATAAG	47.1
C5aR *forward*	ATGCCTGCAGATGGCGTTTA	51.8
C5aR *reverse*	CAGAAACCAAATGGCGTTGAC	52.4
β-Actin *forward*	CGGTCAGGTCATCACTATCG	53.8
β-Actin* reverse*	TTCCATACCCAGGAAGGAAG	51.8

### Statistical Analysis

Experimental groups were coded (named by numbers) for the person who made the statistical analysis to reduce bias. Data analysis was performed using dedicated statistical software (GraphPad Prism 8.0). All results were presented as mean values from individual experimental data, ± standard deviation (SD). Statistical significance analyses were performed on data obtained from at least 3 experiments and 3 technical replicates. Comparisons between groups were performed using two-way analysis of variance (ANOVA) followed by the Bonferroni post-hoc test for multiple comparisons. The data were considered statistically significant at *p*-value < 0.05. Power analysis: The sample sizes for each of the studied groups were determined with the assumptions: 99,5% confidence level, 90% power of the test. With these parameters, the minimum required sample size in each group was 3. The numbers were determined based on preliminary data, assuming that for the complement protein C5 index, which is one of the key proteins in the study, the difference between means will be 30 and the standard deviation will be SD = 12.

## Results

### Architecture of Synaptic Connections

**Fig. 2:**
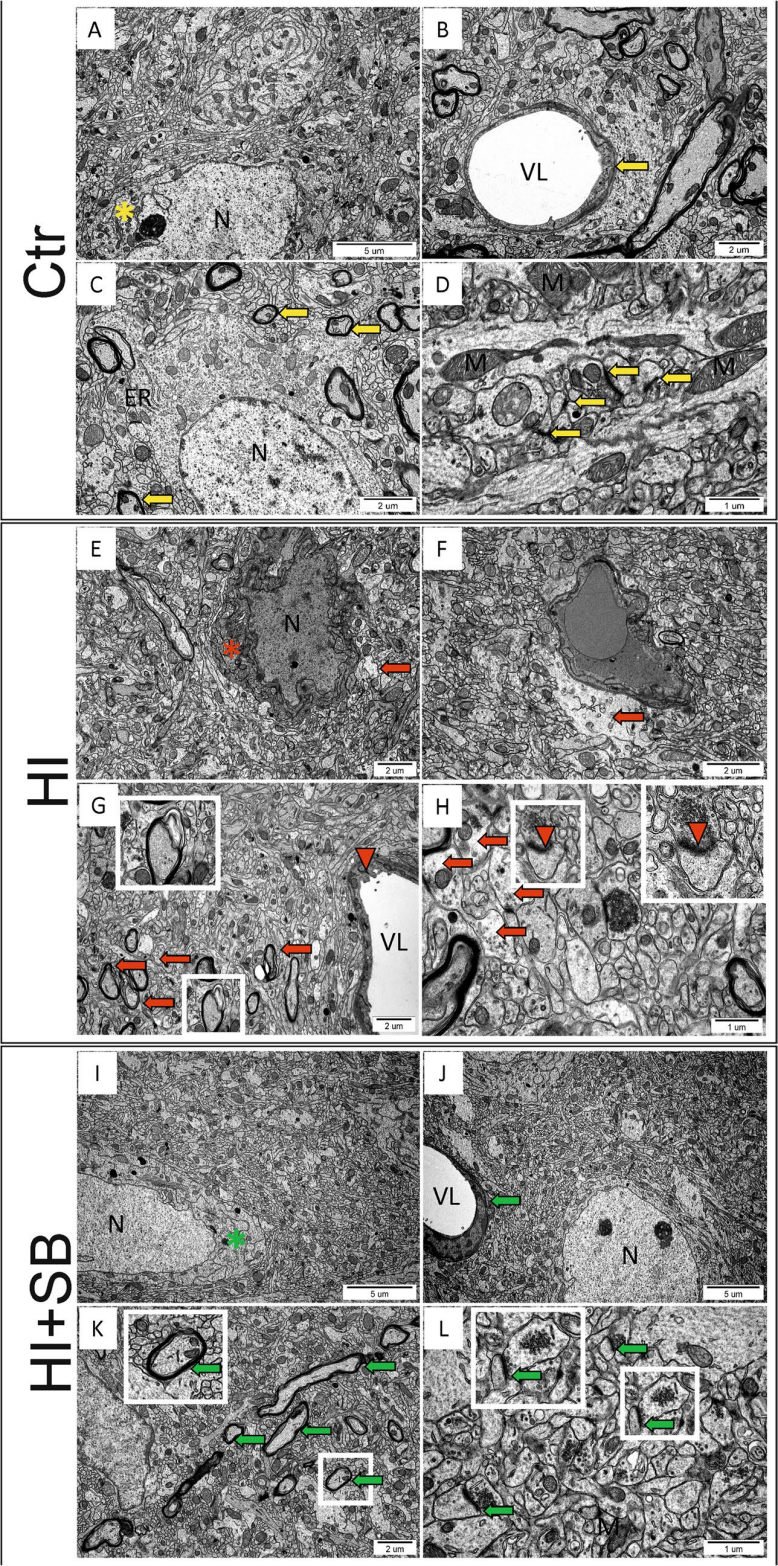
Structure of the synaptic connections. Ultrastructural analysis of frontal cortex from control and HI-induced animals in the presence and absence of sodium butyrate by electron microscopy was performed. Material was collected 1 week after induction of HI. Panels **A**–**D** show the electronographs of control animals. No ultrastructural changes were observed. **A** Bo oedema of neural tissue, yellow *—morphologically unchanged neuron, **B** capillary blood vessel, no perivascular oedema (yellow arrow), **C** compact myelin sheaths (yellow arrows), **D** morphologically unchanged synaptic structures (yellow arrows). Tissue degradation was noted in animals after HI (Fig. 2E-H). **E** Morphological features of oedema of the neural tissue (red arrow), red *—“dark neuron”, **F** morphological features of perivascular oedema (red arrow), **G** degradation of the myelin sheaths (red arrows), endothelial protrusions into vessel lumen (red arrowhead), **H** morphological features of oedema of synaptic endings (red arrows) and blurred synaptic structures (red arrowheads). Neuroprotective effect was observed after sodium butyrate treatment (**I**–**L**). **I** No edema of neural tissue, green *—morphologically unchanged neuron, **J** capillary blood vessel, no perivascular oedema (green arrow); **K** compact myelin sheaths (green arrows), **L** morphologically unchanged synaptic structures (green arrows). These results were interpreted in reference to the control (**A**–**D**). Ctr, control; HI, hypoxia–ischemia; HI + SB, hypoxic-ischemic animals after sodium butyrate treatment; N, nucleus; M, mitochondrium; ER, endothelial reticulum; VL, vessel lumen. Number of biological replicates in each experimental group *n* = 5

In the first step of the study, analysis of frontal cerebral cortex ultrastructure was performed, with an emphasis on synaptic connections using transmission electron microscopy. Material was collected 1 week after surgery procedure from control and HI-treated animals, as well as control and HI-induced animals after treatment with sodium butyrate. HI-treated tissue was ultrastructurally altered (Fig. 2E–H). We observed the damage of HI hemispheres, e.g., edema of neural tissue (Fig. 2E), dark neurons (Fig. 2E, red asterisk (*)), perivascular edema (Fig. 2F, red arrow), endothelial protrusions into vessel lumen (Fig. 2G, red asterisk (*)), stratified, degrading myelin (Fig. 2G, red arrows, white frames), blurred synaptic structures (Fig. 2H, red arrows, white frames), and swelling of synaptic endings (Fig. 2H, red arrows). Sodium butyrate treatment had a neuroprotective effect. Animals treated with SB showed better morphology of neurons (Fig. 2I, green asterisk (*)), less edema of neural tissue (Fig. 2I), no more severe perivascular edema (Fig. 2J, green arrow), normal structure of myelin sheaths in most of the studied tissue (Fig. 2K, green arrows, white frames), and better ultrastructure of synaptic connections (Fig. 2L, green arrows, white frames) in comparison to HI animals non-treated with SB. As we did not notice any alteration of the investigated tissue in Ctr + SB group, we decided not to include it in the TEM panel.

### Loss of Synapses Induced by Neonatal HI

**Fig. 3:**
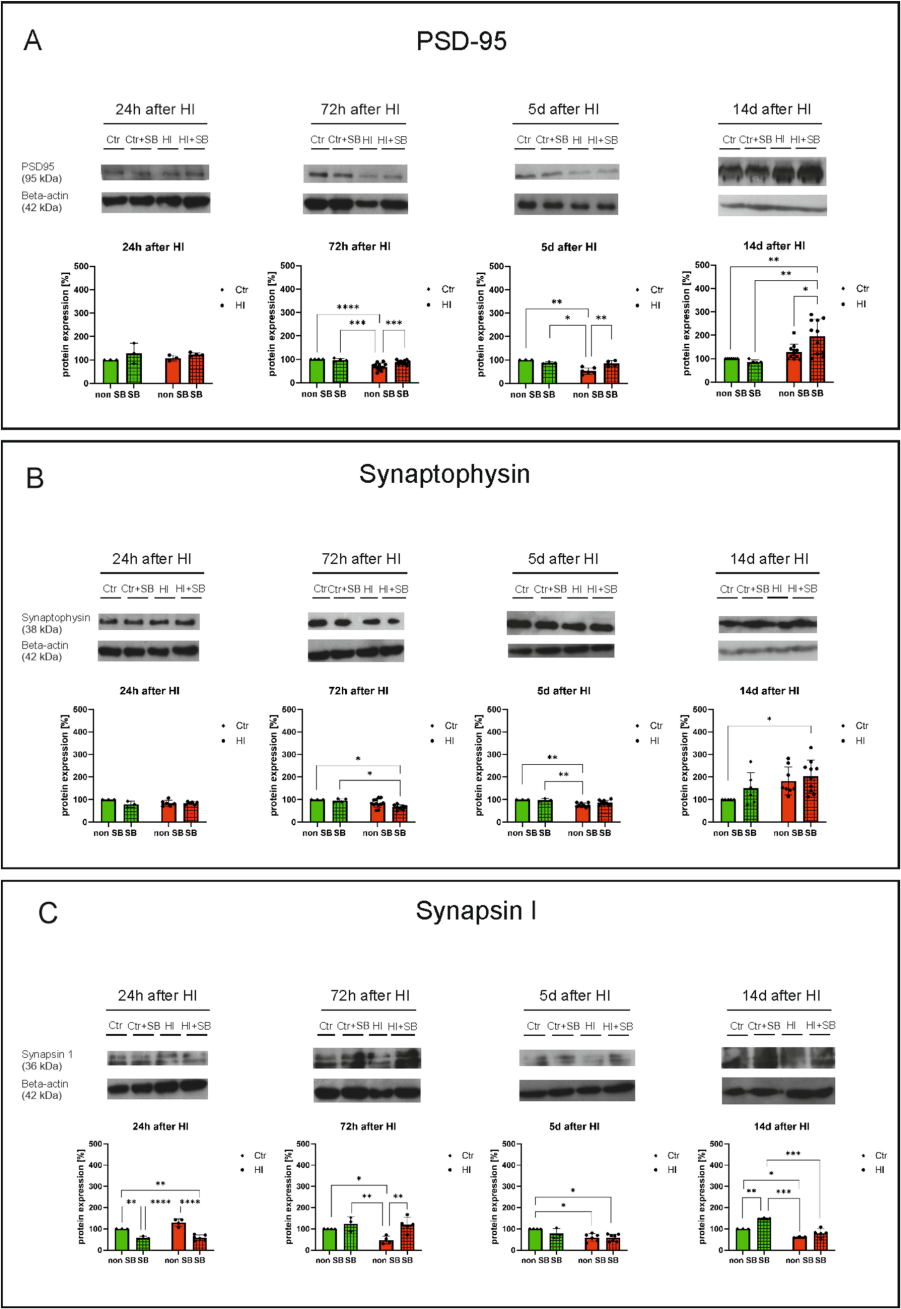
Loss of synapses induced by neonatal HI. Western Blot analysis was performed on material obtained from HI-induced and control animals in the presence and absence of sodium butyrate. Material was obtained 24 h, 72 h, 5 days, and 14 d after induction of HI. **A** Results for PSD-95, **B** results for synaptophysin, **C** results for synapsin I. Ctr, control; Ctr + SB, control in the presence of sodium butyrate; HI, hypoxia–ischemia; HI + SB, hypoxia–ischemia in the presence of sodium butyrate; non-SB, not treated with sodium butyrate; SB, treated with sodium butyrate. The results were obtained by densitometric analysis of the grey levels of the bars expressed as a percentage of the ratio of the optical density (OD) of the Ctr + SB, HI, and HI + SB bars concerning Ctr. Results are presented as mean values ± SD. Number of biological replicates in each experimental group *n* ≥ 3. Statistical analysis: two-way ANOVA with Bonferroni post hoc test: ****p < 0.0001, ***p < 0.001, **p < 0.01, *p < .0.05. Ctr, Ctr + SB – green bars; HI, HI + SB – red bars

In the next step, we performed the experiments to determine the expression of molecules that were components of synaptic membranes, in the presence and absence of sodium butyrate. Western blot analyses were performed to determine the levels of presynaptic proteins-synaptophysin and synapsin I and the postsynaptic protein – PSD-95 in samples obtained from control and HI-induced animals, as well as control and HI-treated animals after sodium butyrate administration. Hypoxia–ischemia decreases the expression of PSD-95 protein in the ipsilateral hemisphere 72 h and 5 days after HI, while after treatment with sodium butyrate, it returns to the level observed for the control. Sodium butyrate also increased PSD-95 protein expression after 14 days in animals after HI compared to the group of animals not treated with this inhibitor (HI + SB vs HI, *p* < 0.05) (Fig. 3A). Moreover, we observed a decreased expression of synaptophysin after HI, but these results did not clearly indicate a protective effect of sodium butyrate. Reversely, a significant decrease in the level of synaptophysin was observed 72 h after HI induction in the group of SB-treated animals. Twenty-first hours after HI we noticed a slight trend in decreased expression of this protein, but SB administration did not affect it. When the survival time of the animals was extended to 5 days, there was a temporary decrease in the expression of this protein in the brains of hypoxic-ischemic animals, with a slight increase in the level of expression after the application of SB, but 14 days after HI induction an increase in the level of synaptophysin was observed, which was further significantly increased after SB treatment (Fig. 3B). Hypoxia–ischemia statistically significantly decreases synapsin I protein expression in the ipsilateral hemisphere 72 h and 14 days after HI, while a significant increase was observed after sodium butyrate treatment. A decrease in synapsin I expression was also observed in the ipsilateral hemisphere 5 days after HI, while the SB administration did not change the level of the synapsin I expression compared to the HI group (HI + SB vs HI) (Fig. 3C).

### Localization of the Complement System Components in the Brain After HI

**Fig. 4:**
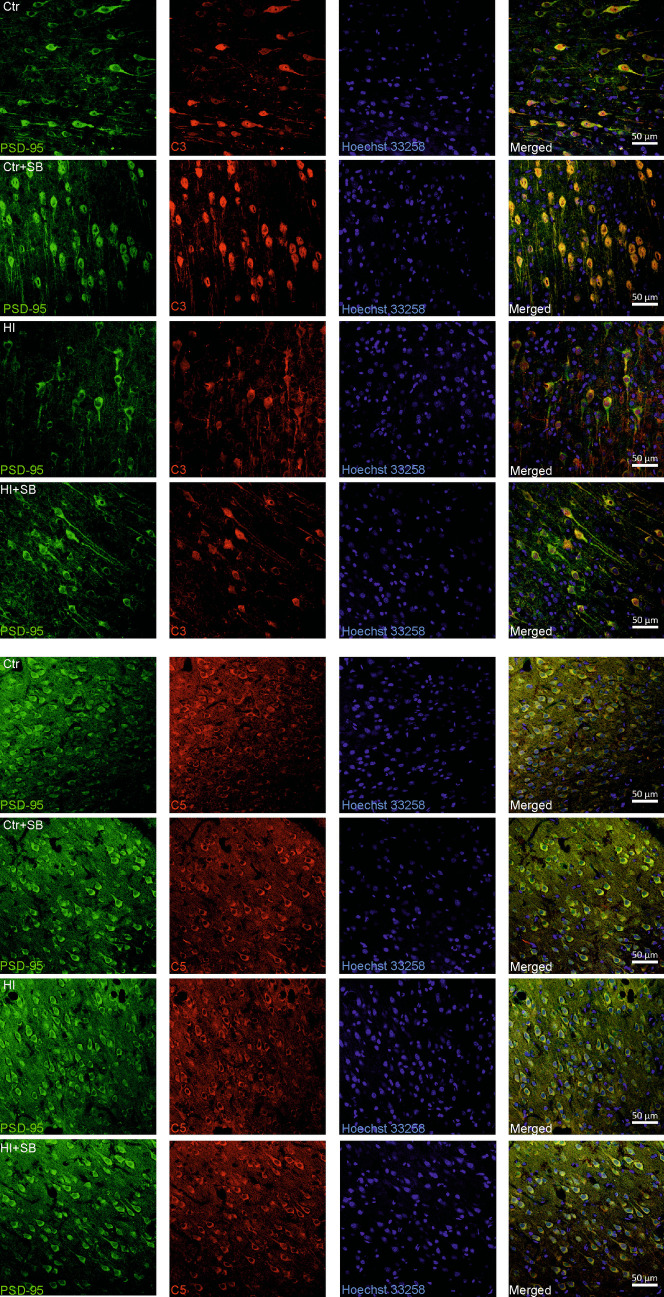
Localization of complement system components in the brain. Double immunohistochemical staining was performed on brain slices of HI-treated animals in the presence and absence of sodium butyrate (2 weeks after HI induction), as well as control animals. Ctr, control; HI, hypoxia–ischemia; Ctr + SB, control animals after sodium butyrate treatment; HI + SB, hypoxic-ischemic animals after sodium butyrate treatment. Color green, PSD-95; red, C3/C5; blue, Hoechst 33,258. Scale 50 μm

The next step of the study was to localize the proteins of the complement system in the brain in the presence and absence of the studied inhibitor. We tested whether complement proteins are located in close proximity to synapses, and whether HI and SB treatment affects their location. To determine the presence of the complement C3 and C5 proteins, double immunohistochemical staining was performed on brain slices obtained from control and HI-treated animals, in the presence and absence of SB. The material was collected 2 weeks after induction of HI. Immunohistochemical results were evaluated on a fluorescence microscope. Zeiss LSM ImageBrowser software was used for image analysis. C3 and C5 proteins of the complement system are present in all areas of the brain, including the cerebral cortex, hippocampus, and striatum, in perisynaptic spaces, in both healthy tissue and tissue injured by a hypoxic-ischemic episode (Fig. 4). Numerous colocalizations of C5 and C3 with PSD-95 proteins confirm that they are located in the immediate vicinity of synapses. However, based on the obtained results, it is not possible to clearly determine whether HI or SB affects the localization of complement proteins.

### Expression of the mRNA of Complement System Proteins

**Fig. 5:**
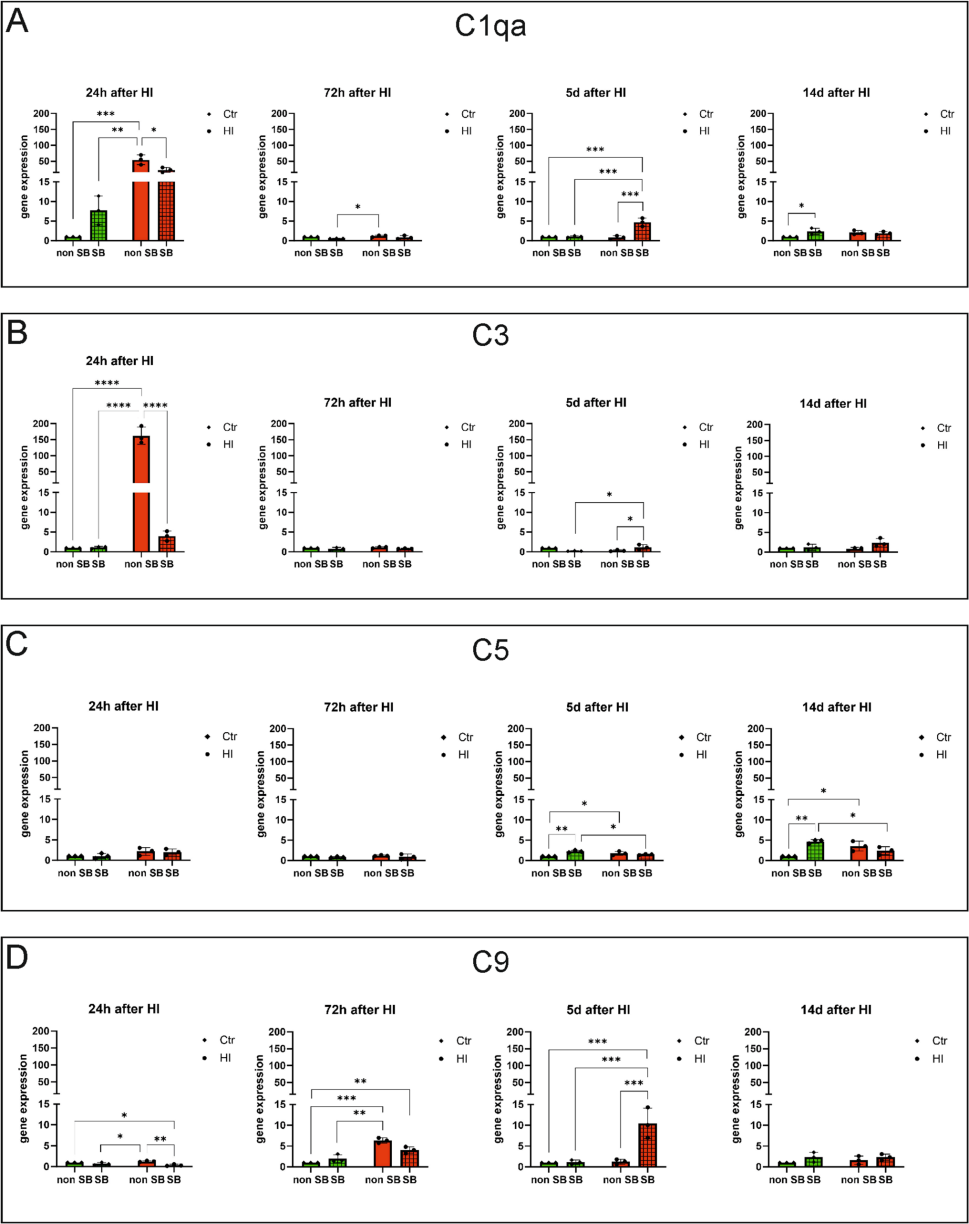
Expression of the mRNA of complement proteins. The qPCR analysis was performed on material from animals induced by HI and control animals, in the presence and absence of sodium butyrate. Material was collected 24 h and 72 h and 5 days and 14 days after HI induction. **A** Results for C1qa, **B** results for C3, **C** results for C5, **D** results for C9. Ctr, control; HI, hypoxia–ischemia; non-SB, not treated with sodium butyrate; SB, treated with sodium butyrate. Results are presented as mean values ± SD, calculated relative to the reference gene actin, and concerning Ctr which is considered 1. Number of biological replicates in each experimental group *n* = 3. Statistical analysis: two-way ANOVA with Bonferroni post hoc test: ****p < 0.0001, ***p < 0.001, **p < 0.01, *p < .0.05. Ctr, Ctr + SB – green bars; HI, HI + SB – red bars

The experiments were performed to determine the expression of the mRNA of molecules that are components of the complement system in the presence and absence of sodium butyrate. To determine the expression levels of mRNA of C1qa, C3, C5, and C9 proteins, qPCR analyses were performed on samples obtained from control and HI-treated animals, as well as control and HI animals treated with sodium butyrate. Material was collected 24 and 72 h, 5 and 14 days after HI induction. After 24 h of the hypoxic-ischemic episode, increased mRNA expression was observed for the C1qa and C3 proteins of the complement system, while after treatment with sodium butyrate the expression was decreased for both components (Fig. 5A, B). The expression of C5 was slightly increased 5 and 14 days (statistically significant results) after HI inductions. Furthermore, after SB administration, expression decreased, but these results were not statistically significant (Fig. 5C). In contrast, after 72 h, increased mRNA expression for the C9 protein of the complement system was noted, which decreased after SB treatment (Fig. 5D). At 5 days and 2 weeks after HI induction, mRNA expression of complement system proteins stayed low in all examined groups, although after 5 days we observed an increase of C1qa, C3, and C9 mRNA expression in the HI + SB group (Fig. 5A, D).

### Expression of the mRNA of Receptors for Proteins of the Complement System

**Fig. 6:**
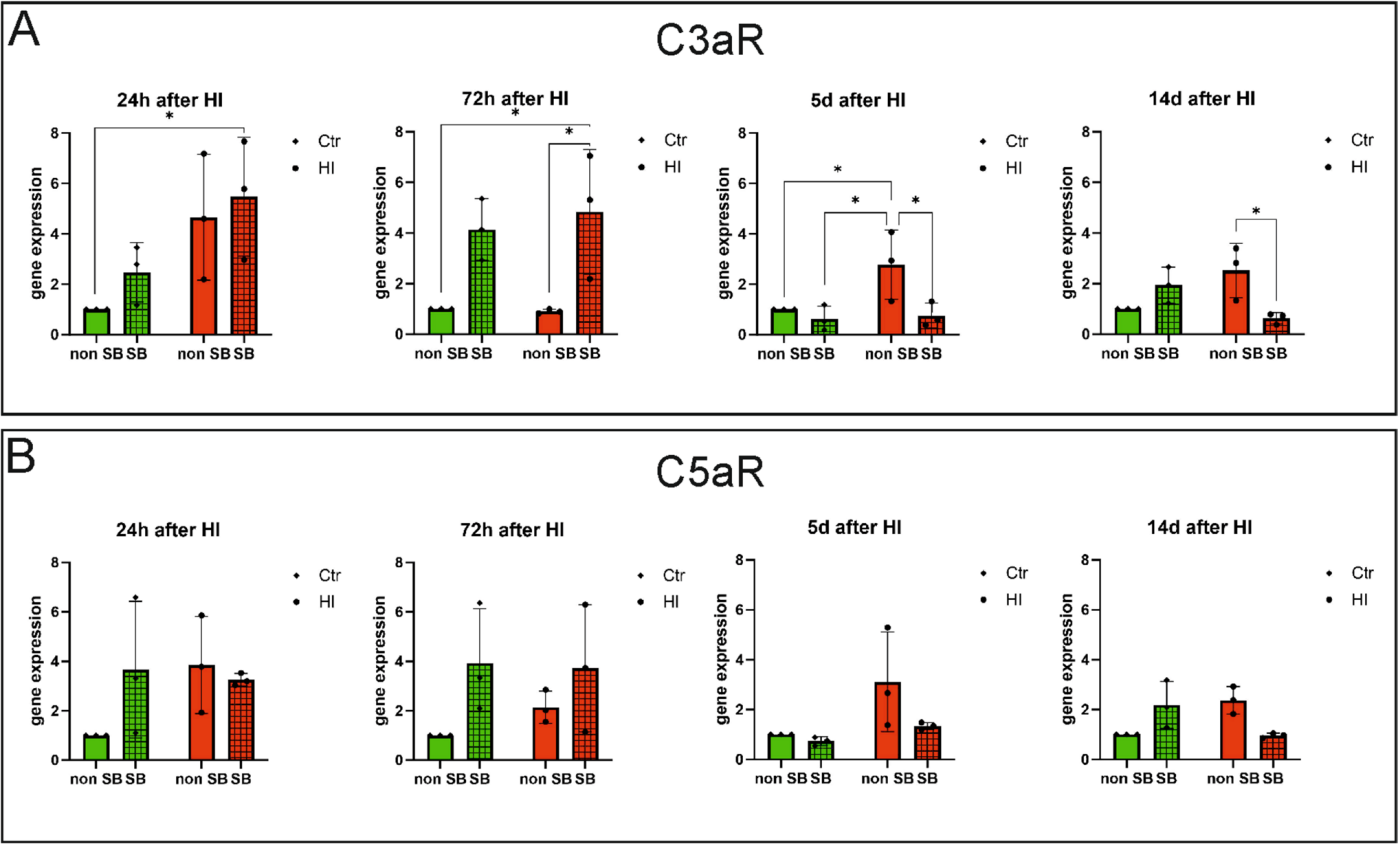
Expression of the mRNA of receptors for complement system proteins. The qPCR analysis was performed on material from HI-induced and control animals, in the presence and absence of sodium butyrate. Material was collected 24 h, 72 h, 5 days, and 14 days after HI induction. **A** Results for C3aR, **B** results for C5aR. Ctr, control; HI, hypoxia–ischemia; non-SB, not treated with sodium butyrate; SB, treated with sodium butyrate. Results are presented as mean values ± SD, calculated relative to the reference gene actin, and concerning Ctr, which is considered 1. Number of biological replicates in each experimental group *n* = 3. Statistical analysis: two-way ANOVA with Bonferroni post hoc test: ****p < 0.0001, ***p < 0.001, **p < 0.01, *p < .0.05. Ctr, Ctr + SB – green bars; HI, HI + SB – red bars

Experiments were performed to determine the expression of mRNA of receptors for complement system proteins in the presence and absence of sodium butyrate. To determine the expression levels of the C3aR and C5aR mRNA, qPCR analyses were performed on samples obtained from control and HI-induced animals, as well as control and HI-induced animals after sodium butyrate administration. The material was obtained 24 and 72 h, 5 and 14 days after induction of HI. Twenty-four hours after the hypoxic-ischemic episode, increased mRNA expression for both studied receptors was noted, while the treatment with sodium butyrate additionally increased expression of C3aR and decreased the expression of C5aR; however, these results were not statistically significant. After 72 h of HI induction, the expression of C5aR increased, while the treatment with sodium butyrate additionally enhanced this expression. The expression of C3aR also increased in samples obtained from HI-induced SB-treated animals (HI + SB group). After 5 days and 2 weeks, increased expression of mRNA of both receptors was still noted in the material from the animals subjected to HI compared to control, while it was decreased after the application of sodium butyrate (Fig. 6A, B).

## Discussion

**Fig. 7:**
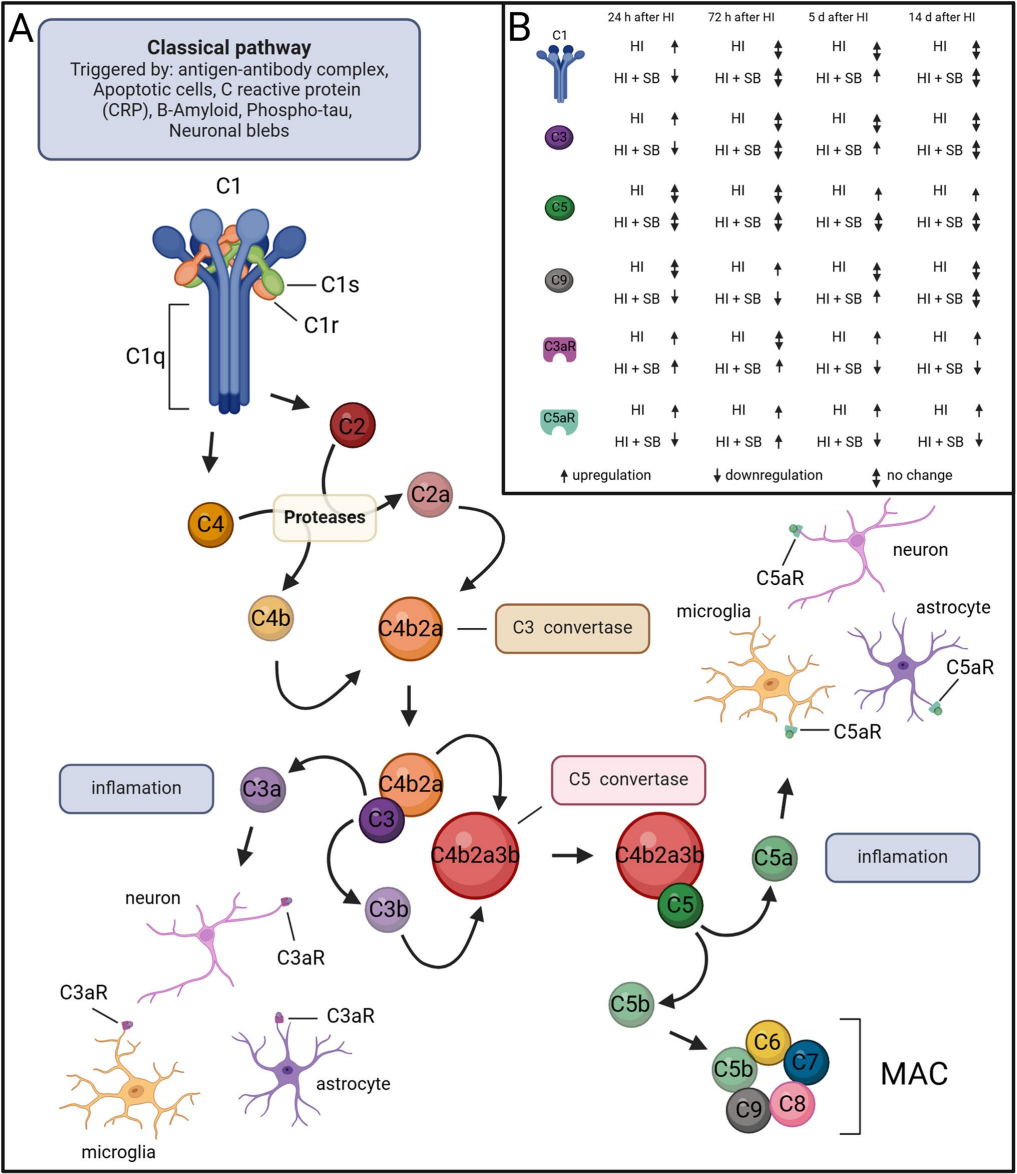
Graphical summary of the study. **A** Schematic diagram of complement system pathway under hypoxia–ischemia. **B** The interaction between sodium butyrate and the complement system. Created with BioRender.com

The present study has shown that treatment with the histone deacetylase inhibitor—sodium butyrate, after neonatal HI—provides neuroprotection. Post-ischemic damage of the nerve tissue with particular emphasis on synapse degradation in the ipsilateral hemisphere was reduced after SB application which was connected with modulation of the inflammatory response and the complement system activity (Fig. 7). Our study has shown decreased levels of presynaptic proteins synapsin I and synaptophysin as well as postsynaptic protein PSD-95 in the ipsilateral hemisphere, indicating synaptic loss after neonatal HI. Moreover, we observed blurred synaptic structures, morphological features of edema of synaptic endings, and dying neurons in the brain cortex and hippocampus after hypoxic-ischemic injury. Synaptic connections are essential for signal transmission between neurons and form the complicated network in the brain [21]. Due to their critical role in the developing nervous system, synapse loss and synaptic dysfunction lead to cognitive, learning, attentional, and motor deficits [22, 23]. Restoring communication between neuronal cells and reconstructing neural circuits are crucial for neurological recovery, and this process depends on the remodeling of synaptic structures and functions to generate normal neural activity and cognitive function [24]. Sodium butyrate treatment in HI animals increased the level of the synaptic proteins, improved tissue ultrastructure, and reduced degradation of the synapses. Our findings remain in general agreement with those previously reported that deacetylase inhibitors (VPA, TSA, SB) are neuroprotective in cerebral injury models in adult rodents [15, 16, 25]. These data also confirm the neuroprotective effect of sodium butyrate after neonatal hypoxia–ischemia in rats described in our previous publications [17, 18, 20]. We observed about 50% weight deficit and severe atrophy of the ipsilateral (injured) hemispheres 14 days after the experimental hypoxia–ischemia compared to the control ones. The administration of sodium butyrate for 5 days following the onset of the hypoxic-ischemic insult appeared to decrease the cerebral damage by preventing atrophy; however, a small degree of asymmetry was still visible [20]. Furthermore, SB application protected against HI-induced loss of neuroblasts and oligodendrocyte precursor cells in two neurogenic regions subgranular zone (SGZ) of the hippocampal dentate gyrus (DG) and the subventricular zone of the lateral ventricles (SVZ) [18, 20]. Our experiments presented in this manuscript demonstrated that SB application has reversed diminished levels of synapse-related proteins caused by HI insult in ipsilateral hemispheres. Synapsin I and synaptophysin are the markers of the presynaptic terminal. These proteins are localized in the membranes of synaptic vesicles and are strongly involved in neuronal development and neurotransmitter release [26, 27]. PSD-95 is the most important and abundant postsynaptic scaffold protein that interacts primarily with synaptic NMDA receptor proteins (primarily NMDARs) and aggregate intracellular signaling as well as is involved in synaptic plasticity regulation [28, 29]. Since these synaptic proteins affect the formation and reconstruction of synapses, their increased expression after SB treatment in hypoxic-ischemic animals may indicate an increased number of synaptic connections and confirm the neuroprotective effect of SB. The molecular mechanisms underlying synaptic remodeling after HI are not fully understood, but recent data suggested that the inflammatory processes may play a significant role in this process.

Inflammation is one of the crucial mechanisms of neuronal injury after neonatal HI. It is characterized by the activation of microglia, migration of peripheral macrophages, the release of cytotoxic and pro-inflammatory cytokines and chemokines, and phagocytosis of injured and uninjured neurons [30–32]. The recent data point to the pivotal role of the complement system in promoting multiple downstream activities that lead to neuroinflammation and neurodegeneration [13]. Our present study demonstrated that complement proteins (C3 and C5) colocalized with PSD-95 protein in the cerebral cortex, hippocampus, and striatum after hypoxic-ischemic episode. These results indicate that the complement system may be involved in the post-ischemic degradation of synaptic connections in immature animals. Moreover, we have shown that neonatal hypoxia–ischemia-induced mRNA expression of the complement proteins C1q, C3, C5, and C9 in the ipsilateral (injured) hemisphere, although the activation pattern of individual complement proteins varies. The complement C1q and C3 were activated strongly 24 h after HI, and after that time point, their activity decreased to the value observed in control animals. Post-ischemic activation of C5 and C9 was much weaker and occurred at later time points after HI.

Post-ischemic upregulation of C1q and C3 was also observed by other researchers in models of transient MCAO in adult animals [4, 33, 34] and after experimental neonatal hypoxia–ischemia [10, 35–39]. Data from human patients who died from a stroke showed a prominent deposition of complement C1q, C3c, C4d, and C9 components in the ischemic brain [40]. The complement activation proteins, C5a and C3a, present in the serum of stroke patients have been correlated with the severity of the pathology and its symptoms [40, 41]. Additionally, few available clinical studies conducted on neonates affected by HIE also indicated activation of the complement system in their brains and/or cerebral spinal fluid (CSF) [11, 12]. The results of the studies cited above clearly indicate that the complement system plays a key role in the pathogenesis of post-ischemic brain injury. Complement activation after brain ischemia is primarily mediated by circulating natural antibodies (IgM) which are binding to neo-antigens expressed on the surface of hypoxia-stressed endothelial cells [42]. Complement proteins pass through the ischemia-damaged blood–brain barrier to the brain parenchyma, although they can be also locally produced in the CNS, mainly by glial cells and together with other proinflammatory cascades contribute to secondary injury after ischemia. Furthermore, post-ischemic complement activation promotes neuronal stress by tagging live neurons for uptake by microglia and reduces the probability of recovery of these neuronal cells, which contributes to the loss of synaptic networks [43]. Recent studies indicate that inhibition of inflammation can promote neuroprotection and has potential for use in the clinical treatment of ischemic brain injury in neonates [44, 45]. However, a therapeutic strategy based on complement inhibition in the developing brain is tricky because complement takes part not only in inflammatory processes, but also in brain development and tissue regeneration. Perhaps because of that, the results of studies concerning inhibition of the complement system after neonatal HI are inconclusive. The results of our experiments demonstrated that neonatal HI strongly increased the expression of C1q and C3 mRNA, and the SB administration decreased its activation, resulting in neuroprotection. Our findings are in agreement with the study performed by Ten et al. [36], which showed that inhibition of the classical C1q-dependent activation pathway after HI had a neuroprotective effect. It is worth noting that in neonatal rodents, unlike in adults, the lectin and alternative pathways are underdeveloped; thus, complement is mainly activated via the classical pathway. This study revealed that deletion of C1q in hypoxic-ischemic mice reduced brain damage and exhibited significantly fewer neurofunctional deficits compared to wild-type controls. The neuroprotective effect of C1q deletion was correlated with the reduction of circulating neutrophils which were strongly activated after HI in WT animals [36]. Moreover, the same study revealed a decreased deposition of C3 in the ischemic brains of C1q(-/-) mice compared with WT animals, which may suggest that C3 activation plays an important role in brain damage after neonatal hypoxia–ischemia. The beneficial effect of classical complement pathway inhibition after neonatal HI was strongly supported by Shah et al. [42]. The authors showed that therapeutic effect of treatment with hypothermia was associated with decreased expression of C1q and C3 in rats’ brains after HI, lower deposition of C3 and C9 on microglia and neurons, and reduced C1q binding on cells undergoing apoptosis [42].

Several studies have established the receptor for complement molecule C3a (C3aR) as a promising therapeutic target after cerebral ischemia. Increased expression of C3aR in brain tissue has been observed in various murine models of transient and permanent MCAO [46–48]. Administration of C3aR antagonist resulted in a reduction of post-stroke tissue infraction by inhibiting the recruitment of neutrophils to the ischemic zone [49]. On the other hand, a set of experiments showed that signaling through C3aR has a neuroprotective effect after neonatal hypoxia–ischemia. Intranasal administration of C3a after hypoxia–ischemia reduced neurodegeneration and reactive gliosis in the hippocampus and ameliorates HI-induced cognitive impairment [37, 50, 51]. The other study has shown that C3a receptor deficiency decreased axonal sprouting and plasticity in the peri-infarct cortex. Intranasal treatment of wild-type mice with C3a robustly increased synaptic density and improved functional impairment after photothrombotic stroke. In addition, C3aR deficiency was associated with reduced expression of synapsin I, a structural element of presynaptic terminals and a marker of synaptic plasticity, and VGLUT1, a presynaptic marker of the majority of glutamatergic synapses [52]. The other study showed that therapeutic hypothermia increased the level of C3a and its C3aR receptor in the brains of neonatal rats after HI, which contributed to decreased inflammation and tissue damage [38]. These reports have suggested that C3a/C3aR signaling plays a key role in the restoration of neuronal functions and tissue regeneration after ischemia. Also, in our present study, we investigated the influence of hypoxia–ischemia and SB applications on the complement receptor C3aR in the brains of rat neonates. The results of this experiment have shown increased expression of C3aR in almost all investigated time points after ischemia, however, mainly without statistical significance. The effect of sodium butyrate was different depending on the time after induction of hypoxic-ischemic damage. In the early time points after HI (24 h and 72 h), sodium butyrate application slightly increased the expression of C3aR, whereas in the later time points (5 and 14 days after HI) SB reduced the mRNA expression of this receptor in the ipsilateral hemisphere in comparison to the non-treated HI animals. Presumably, the biphasic response to sodium butyrate after neonatal hypoxia–ischemia may indicate that in the acute phase, increased expression of C3aR after SB application may decrease post-ischemic brain damage, while in the later phase after HI, decreased expression of this receptor may promote neuro-repair processes.

The next step in activation of the classical complement pathway through C1 is cleavage of C5 which generates soluble C5a and membrane-bound C5b. Proinflammatory C5a acting via C5aR contributes to the recruitment and activation of leukocytes and accelerates postischemic brain damage. Protein C5b together with C9 initiates assembly to the membrane attack complex (C5b-9, MAC) that can cause direct cell lysis, but that can also stimulate cells to release inflammatory molecules [13]. The results of our study demonstrated that neonatal HI enhanced C5 mRNA expression 5 and 14 days after HI in the ipsilateral hemisphere and application of SB to hypoxic-ischemic animals prevented an increase of C5 expression. Additionally, we observed increased expression of C5aR in all investigated time points after HI, however without statistical significance. The effect of sodium butyrate was biphasic, 72 h after HI we observed enhanced C5aR mRNA expression, and in a later phase after the injury SB administration decreased the level of this receptor in the ipsilateral hemispheres. In general, the functional role of C5 in ischemic brain damage is unclear and strongly depends on the time and severity of the injury [53]. Mocco et al. [34] reported that C5-deficient mouse cohort did not exhibit neuroprotection 24 h after focal cerebral ischemia. The neurological score and neuronal loss in C5 knock-out mice did not differ compared to WT mice subjected to ischemia. Conversely, an experiment conducted by Arumugam et al. [54] demonstrated that 72 h after permanent stroke induction C5-/- mice had better functional outcomes and reduced size of brain infarction compared with WT littermates. Moreover, inhibition of the C5a-C5aR axis by using the C5 antibody diminished cerebral lesion and improved motor functions in a rat model of stroke [55], and treatment with C5a receptor antagonist—PMX53 significantly decreases ischemia-induced apoptosis of primary mouse cortical neurons subjected to oxygen–glucose deprivation (an in vitro model of hypoxia–ischemia) [56]. The elevation of C5a and C5aR after neonatal hypoxia–ischemia was also observed by Shah et al. [38] and treatment with therapeutic hypothermia decreased the level of both proteins, resulting in neuroprotection. As mentioned above activation of the C5a–C5aR axis after ischemia exacerbates brain injury by enhancing the release of inflammatory cytokines and chemokines from activated microglia and astrocytes, which induce chemotaxis and activation of granulocytes and leukocytes and leads to inflammation [57]. The studies have mostly shown the positive results of inhibition of C5a–C5aR signaling in an acute phase of ischemic brain injury. However, the lack of C5aR signaling in the post-acute period can be detrimental to recovery. Bernnan et al. [58] reported that prolonged interruption of C5aR signaling following spinal cord injury leads to impaired glial scar formation limiting border around the lesion core, as evidenced by larger lesion volumes and poorer neurological recovery. The biphasic response of C5aR to SB treatment revealed in our study is not consistent with the result obtained by Bennan’s group. The sodium butyrate used in our experiments resulted in a modest and statistically insignificant decline in C5aR expression in a later phase of hypoxic-ischemic injury, which indicated that decreased level of this receptor 5–14 days after HI may have a positive effect. This discrepancy may be due to the use of different animal models and the various methods of modulation/inhibition of C5aR signaling.

The last component generated by classical complement pathway activation investigated in our study was C9. Analysis of the C9 protein showed a significant increase in mRNA expression in the ipsilateral hemisphere, but only 72 h after hypoxia–ischemia induction. The application of SB reduced the level of C9 in the early phase after HI; however, at a 5-day time point, we observed upregulation of C9 mRNA expression. The effect of SB on C9 was similar to that observed with C1q and C3. We hypothesize that decreased expression of C9 after SB application may reduce post-ischemic brain damage in the early phase after HI, while in the later phase, enhanced expression of this protein may promote repair processes (e.g., by tagging dead neurons to remove from the tissue by microglia). The complement component C9 is the major cytolytic protein that binds to the assembly membrane attack complex (MAC) and leads to the destruction of the target membranes. Studies performed by Schultz et al. [11] on post-mortem cerebral tissue of newborn infants who suffered from hypoxic–ischemic encephalopathy demonstrated that C9 was deposited on neurons, mostly in the hippocampus. Moreover, C9 was deposited on injured neurons that demonstrated morphological characteristics of apoptosis (e.g., nuclear fragmentation) suggesting the involvement of this complement protein in the modulation of post-ischemic neuronal apoptosis [11]. In neonatal rats subjected to an experimental hypoxia–ischemia, systemic complement inhibition by cobra venom factor (CVF) reduced the deposition of C9 on neurons and decreased ischemic cerebral infraction [10]. On the other hand, the administration of C9 to neonatal rats’ augments brain damage induced by hypoxia–ischemia [59].

## Conclusion

Our study demonstrated that the neuroprotective effect of sodium butyrate may be related to modulation of complement activity after experimental hypoxic-ischemic brain injury. However, our study has some limitations. It should be emphasized that although the protective effect of SB on post-ischemic synapse degradation was connected with significant changes in complement proteins and respective receptors’ expression, these associations do not prove causality. It is possible, that modulation of mRNA expression of complement proteins is not a direct consequence of SB action, but is a result of its nonspecific neuroprotective effects. Continuing research about the mechanisms of SB action may provide a better understanding of responses to the inhibition of histone deacetylases in the neonatal hypoxic-ischemic brain. Furthermore, a better understanding of the time course of complement involvement after birth asphyxia may identify a therapeutic window during which complement suppression will improve outcomes.

## Data Availability

The datasets generated during and/or analyzed during the current study are available from the corresponding author on reasonable request.
